# ALDH Maintains the Stemness of Lung Adenoma Stem Cells by Suppressing the Notch/CDK2/CCNE Pathway

**DOI:** 10.1371/journal.pone.0092669

**Published:** 2014-03-26

**Authors:** Zhongjun Li, Yang Xiang, Lixin Xiang, Yanni Xiao, Fengjie Li, Ping Hao

**Affiliations:** 1 Department of Oncology, The Second Affiliated Hospital, Third Military Medical University, Chongqing, China; 2 Department of Blood Transfusion, The Second Affiliated Hospital, Third Military Medical University, Chongqing, China; Cincinnati Children's Hospital Medical Center, United States of America

## Abstract

**Objective:**

To evaluate the expression of ALDH1A1 in lung adenoma stem cells (LASCs) and maintenance of their stemness through the Notch pathway.

**Methods:**

LASCs (A549s) were isolated from lung adenoma cells (A549) and identified by their coexpression of CD133 and CD326 and their capacity formulti-directional differentiation. Expression of ALDH1A1 in A549 and A549s cells were evaluated by Real-time PCR. Effects of ALDH1A1 upregulation in A549 cells and its downregulation in A549s cells on the clonogenicity and cell cycle were assessed by colony-forming unit assay. Moreover, the effects of ALDH1A1 on the Notch pathway, and thus on the cell cycle, were studied.

**Results:**

A549s cells were successfully isolated and identified.ALDH1A1expression was significantly higher in A549s than in A549 cells. Clonogenicity was significantly decreased in A549s cells treated with ALDH1A1 siRNA. Duration of the G1 stage of the cell cycle increased after ALDH1A1 was overexpressed, or decreased with ALDH1A1 siRNA. ALDH1A1, Notch1, −2, and −3, CDK2, and CCNE1 expression levels were higher in A549s cells than in A549 cells. Expression of Notch1, −2, and −3, CDK2, and CCNE1 was significantly decreased by upregulation of ALDH1A1 in A549 cells, but increased by its interruption in A549s cells. When Notch3 or CDK2 expression was downregulated, the expression levels of ALDH1A1, Notch1, −2, and −3, CDK2, and CCNE1 were reduced in all cell types.

**Conclusion:**

ALDH1A1 expression improved clonogenicity and inhibited the cell cycle, maintaining the stemness of the A549s cells; this may involve suppression of the Notch/CDK2/Cyclin pathway.

## Introduction

Cancer stem cells (CSCs) are special subpopulations that contain stemcell-specific characteristics, such as self-renewal, unlimited proliferation, maintenance at low differentiation states, and resistance to radiotherapy and chemotherapy, which maybe partially responsible for the proliferation, metastasis and therapy resistance of cancer cells [1]. Because of the capacity of self-renewal, CSCs has infinite proliferation ability and high tumorigenicity. This characteristic may be considered as representing the stemness of CSCs [2]. The mechanism by which the stemness of CSCs promotes their resistance to chemotherapeutic agents and radiotherapy remains unclear. Studies on the maintenance and regulation of stemness are critical for the understanding and control of cancer cells.

Lung adenoma has a relatively high malignancy rate, with rapid progression, high recurrence rate, and resistance to radiotherapy and chemotherapy. Lung adenoma stem cells (LASCs) were confirmed to have significant involvement in the clinical features of lung adenoma [3]. Aldehyde dehydrogenase (ALDH) is considered a biomarker for stem cells [4] and its expression is also thought to closely correlate with the stemness of CSCs [5]. Recently, ALDH1A1 has been considered to have prognostic significance in early stage non-small cell lung cancer, and its effects on lung CSCs have been noticed [6]. However, the pathways by which ALDH affects CSC stemness remain to be identified.

ALDH1A1 has also been reported to play a role in notch signaling in LASCs [7]. Notch can regulate the Akt signaling pathway and eventually affect cell cycle regulatory proteins including cyclin (CCN) and cyclin-dependent kinase 2 (CDK2) [8]. In this study, we evaluated the effects of ALDH1A1 on the stemness of LASC, as well as its potential mechanism, by suppressing the notch pathway.

## Materials and Methods

### Isolation and identification of A549s with induced differentiation

LASCs were isolated from the human LASC line A549 (purchased from American Type Culture Collection), as previously described [9]. Cells positive for both CD133 and CD326 in excess of 80%, as determined by flow cytometry, were preliminarily confirmed as LASCs (A549s) [10]. The isolated A549s cells were cultured in complete culture medium: DMEM/F12 culture medium containing insulin (5000 ng/ml), epidermal growth factor (20 ng/ml), and basic fibroblast growth factor (bFGF; 10 ng/mL), with 5% CO_2_ saturated humidity at 37°C.Further confirmation was obtained from two induced differentiation procedures. Firstly, in order to induce differentiation into cancer cells, cells were cultured in 90% RPMI1640 culture medium containing 10% fetal bovine serum. Secondly, in order to differentiate cells into endothelial cells, cells were cultured in M199 medium containing 2% fetal bovine serum, 50 μg/L vascular endothelial growth factor (VEGF) 165 and 10 μg/L bFGF. Both differentiation procedures were performed in the presence of 100 mg/L penicillin and 100 U/mL streptomycin sulfate, with 5% CO_2_ saturated humidity at 37°C for two weeks.

### Upregulating ALDH1A1 in A549 by slow virus transfection

The LV-TOPO vector was double enzyme digested with *Eco*RV and *Xho*I and the CDS region of human ALDH1A1 was inserted. The target vector was confirmed using enzyme analysis and DNA sequencing, and was then transfected into A549 cells. Successful transfection of the target cells was confirmed using RT-PCR. The sequences of the primers used were as follows:ALDH1A1-R, 5′-CCCGCTCAACACTCCTTCGA-3′; ALDH1A1-F, 5′-CGGGAAAAGCAATCTGAAGAGG-3′.

### SiRNA-mediated downregulation of *ALDH1A1*, *Notch3*,and *CKD2*in A549 cells

A549s cells (1.5×10^5^ cells/well) were cultured with serum-free culture medium containing 0.1 μM double-stranded siRNA and 0.2% Lipofectamine 2000 for 6 h. Subsequently, cells were cultured in complete culture medium for 48 h, before total mRNA was extracted for further studies. The sequences for downregulating ALDH1A1, Notch3, and CKD2 in A549s cells were as follows: ALDH1A1 siRNA-1, 5′-GGCTAAGAAGTATATCCTT-3′;*ALDH1A1* siRNA-2, 5′-CAAATCATTCCTTGGAATT-3′;*ALDH1A1* siRNA-3, 5′-TGATTTAATCGAAAGAGAT-3′; *Notch3* siRNA-1, 5′-CAATAAGGACATGCAGGAT-3′; *Notch3* siRNA-2, 5′-AATGCCAACTGAAGAGGAT-3′; *Notch3* siRNA-3, 5′-CGGTAGTAATGCTGGAGAT-3′; *CDK2* siRNA-1, 5′-CTGATTACAAGCCAAGTTT-3′; *CDK2* siRNA-2, 5′-GACCCTAACAAGCGGATTT-3′; *CDK2* siRNA-3, 5′-ATGCCTGATTACAAGCCAA-3′. The most effective of each of the three siRNA sequences was selected for further studies.

### Expression of ALDH1A1, Notch1, Notch2, Notch3, Notch4, CKD2, and CCNE1 with fluorescent quantitation PCR

The real-time PCR primer sequences were as follows: ALDH1A1-R, 5′-CCCGCTCAACACTCCTTCGA-3′;ALDH1A1-F, 5′-CGGGAAAAGCAATCTGAAGAGG-3′; NOTCH1-R, 5′-GCGGCTCACGGTCTCACTC-3′; NOTCH1-F, 5′-TCCGCGGCTCCATCGTCTACC-3′; NOTCH2-R, 5′-GGATCGGGGTCACAGTTGTCA-3′; NOTCH2-F, 5′-ATGGGCAGTGTGTGGATAAAGTC-3′; NOTCH3-R, 5′-GCCGCAGTCATCCTCATTAATCT-3′; NOTCH3-F, 5′-GGGACCTGCCGTGGCTATATG-3′; NOTCH4-R, 5′-GCCCCACAGCCACCACTCAG-3′; NOTCH4-F, 5′-CCCCACTAGGCGAGGACAGC-3′; CDK2-R, 5′-GCCGAAATCCGCTTGTTAGGG-3′; CDK2-F, 5′-GTGGGCCCGGCAAGATTTTAG-3′; CCNE1-R, 5′-GCTCCCCGTCTCCCTTATAACC-3′; CCNE1-F, 5′-TCGGCCTTGTATCATTTCTCGTC-3′; β-actin-R, 5′-ACCCCGTGCTGCTGACCGAG-3′; β-actin-F, 5′-TCCCGGCCAGCCAGGTCCA-3′.Real-time PCR was then performed with 2 μL of the cDNA product plus 10 μL of 2×SYBY Green PCR, 0.3 μL of 10 μmol/L of specific primers, and water to obtain a final volume of 20 μL. The PCR conditions were as follows: 94°C for 5 min and 40 cycles of a three-step PCR (94°C for 30 s, 60°C for 30 s, 72°C for 40 s).

### Clonogenicity evaluation of A549s cells and A549s+ALDH1A1 siRNA

A549s cells (500 cells/well) treated with (A549s+ALDH1A1siRNA) or without ALDH1A1siRNA were cultured in 2 mL complete culture medium with 5% CO_2_ saturated humidity at 37°C. The clonogenicity of the cells was evaluated two weeks later.

### Flow cytometric cell cycle analysis of A549, A549+ALDH1A1, A549s, and A549s+ALDH1A1siRNA

A549 and A549s cells, treated with (A549+ALDH1A1 and A549s+ALDH1A1siRNA, respectively) or without ALDH1A1siRNA, were washed with 1×PBS and then fixed with ice-cold 70% ethanol. Samples were again washed with 1×PBS and then stained with propidium iodide (60 μg/mL, Sigma) containing RNase (100 μg/mL; Sigma) for 30 min at 37°C, after which flow cytometry analysis (Beckman Coulter, MoFlo XDP) was performed. The data were from three replicates and presented as mean ± S.D. A P<0.05 was considered statistical significance.

## Results

### Isolation and identification of A549s after induction of differentiation

**Figure 1:**
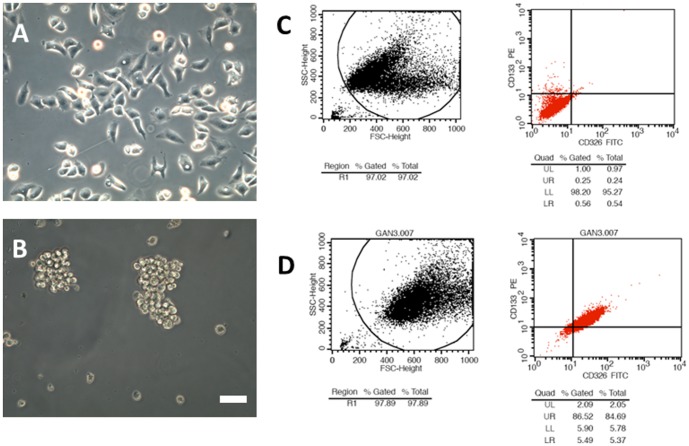
The isolation and identification of A549s. **A** A549 cells seen under light microscopy. **B** A549s cells seen under light microscopy (Scale bar = 50 μm). **C** Expressions of CD133 and CD326 in A549 cells. **D** Expressions of CD133 and CD326 in A549s cells (>80%).

A representative image of A549 cells is present in Fig. 1A. A549s cells (Fig. 1B) were successfully isolated from A549 cells. The expression levels of CD133 and CD326 in A549 cells are shown in Fig. 1C. Flow cytometry was used to isolate and identify A549s cells with expression of these markers at levels exceeding 80% (Fig. 1D).

**Figure 2:**
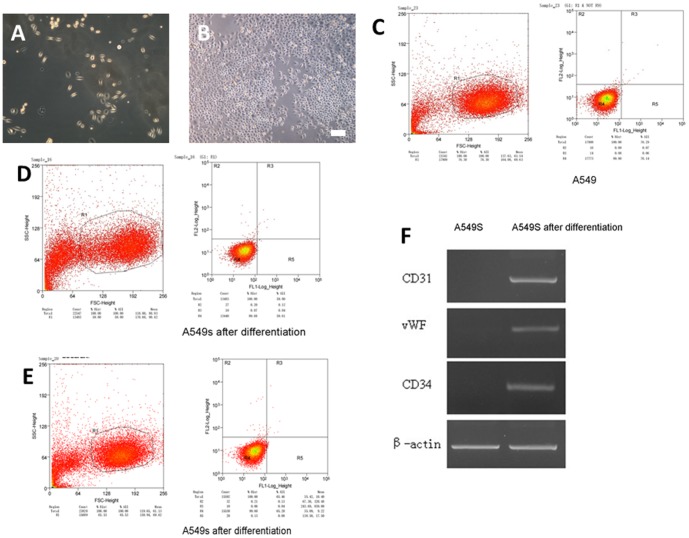
Induced-differentiation of A549s cells. **A** Differentiation of A549s cells into cancer cells at 1 week, seen under light microscopy. **B** Differentiation of A549s cells into endothelium at 1 week, as seen under light microscopy. **C** Expressions of CD133 and CD326 in A549 cells. **D** Expressions of CD133 and CD326 in A549s cells after differentiation. **E** Expressions of CD133 and CD326 in A549s cells after differentiation into endothelium. **F** Expressions of markers CD31, vWF, and CD34 in A549s and differentiated A549s cells. 1. A549s cells 2. Post-differentiated A549s cells. Scale bar = 100 μm in A and B.

A549s cells were successfully differentiated into cancer cells at 1 week (Fig. 2A), whereupon these cells stopped expressing CD133 and CD326 (Fig. 2D). Moreover, A549s cells were successfully differentiated into endothelium (Fig. 2B), marked by expression of CD31, vWF, and CD34 (Fig. 2F). and loss of CD133 and CD326 (Fig. 2E).

### Expression of ALDH1A1 in A549s and its effects on stemness

**Figure 3:**
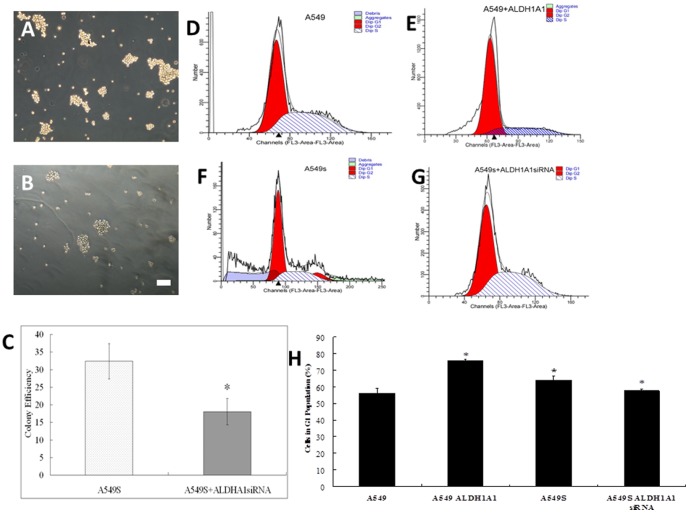
Clonogenicity and cell cycle evaluation. **A** Clonogenicity of A549s cells. **B** Clonogenicity of A549s cells treated with *ALDH1A1* siRNA (A549s+ALDH1A1siRNA). **C** Clonogenicity contrast study. **D** Cell cycle in A549 cells. **E** Cell cycle in A549+ALDH1A1 cells. **F** Cell cycle in A549s cells. **G** Cell cycle in A549s treated with *ALDH1A1* siRNA (A549s+ALDH1A1siRNA) **H** Cells in G1 phase Scale bar = 100 μm in A and B.

Images of clonogenicity of A549s cells (Fig. 3A) and A549s cells treated with *ALDH1A1* siRNA (Fig. 3B) are present. The expression of ALDH1A1 in A549s cells(set at a relative expression of 1) was significantly higher than that of A549 cells (relative expression of 0.0016). The clonogenicity of the cells decreased significantly when ALDH1A1siRNA was applied to A549scells (32.0±3.7 v.s 18.0±3.0, Fig. 3C). Regarding the cell cycle, 56.29±2.66% of A549 cells remained in G1 phase (Fig. 3D), and over expression of ALDH1A1 significantly increased A549 cells remained in G1 phase (75.61±0.94%, Fig. 3E). For A549s cells, 64.06±2.30% cells remained in the G1 stage (Fig. 3F), and ALDH1A1 siRNA application significantly decreased G1 polulation to 57.61±1.29% (*P*<0.05) (Fig. 3G‵H).

### Effects of modulation of expression ofALDH1A1, Notch1, −2, and −3, CDK2, and CCNE1

**Figure 4:**
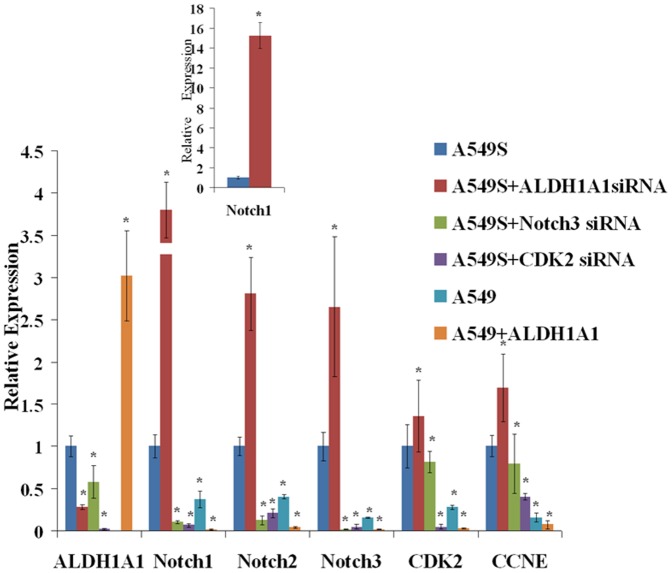
Effect of *ALDH1A1* on the Notch/CDK2/CCNE pathway. The expression levels of these genes in A549s were set at 1, and the relative expression levels in the other cells are expressed by their ratio to that in A549s cells.

Expression levels of ALDH1A1, Notch1, −2, and −3, CDK2, and CCNE1 in each group were quantitative analyzed and data are present in Fig. 4. Expression levels of ALDH1A1, Notch1, −2, and −3, CDK2, and CCNE1 in A549s cells were all significantly higher than those in A549 cells. Upregulation of ALDH1A1 in A549 cells significantly decreased expressions of Notch1, −2, and −3, CDK2, and CCNE1.Depletion of ALDH1A1 expression in A549s cells significantly increased expressions of Notch1, −2, and −3, CDK2, and CCNE1. When Notch3 or CDK2 was downregulated, the levels of ALDH1A1, Notch1, −2, and −3, CDK2, and CCNE1 were inhibited in all cell types (Fig. 4).

## Discussion

Lung adenocarcinoma is one of the most severe malignant tumors, causing a linear multistep progressive disease that may arise from any of several different molecular pathways [11]. Even after surgery, chemotherapy, and/or radiotherapy, the 5-year survival rate of lung adenocarcinoma is still less than 15% [12]. Many reports have demonstrated that tumors relapse from residual cancer cells after therapy, mainly from CSCs. Based on their self-renewal, unlimited proliferation, and multi-directional differentiation, CSCs are considered to be responsible for many special characteristics of cancers, including lung cancers. CSCs were first found in leukemia, and have since been confirmed present in many malignant tumor types [13]. Leukemia stem cells were shown to be resistant to apoptosis after exposure to irradiation or chemotherapeutic drugs due to their arrest in G0/G1, and these quiescent stem cells in cases of minimal residual cancer were responsible for the cancer relapse [14]. CSCs in lung cancers have also been observed [15]. Their unlimited self-renewal, chemoresistance, and ability to switch to cancer cells make clinical treatment difficult. It is therefore important to clarify the mechanism of action of CSCs, their function, and effects on cancer proliferation, invasion, and chemoresistance. In this study, we first successfully isolated A549s cells from A549 cells. In addition to the expression of the marker proteins CD133 and CD326, A549s cells were further confirmed via their capacity for multi-directional differentiation into lung cancer cells and endothelial cells. Moreover, our results showed that A549s cells express a high level of ALDH1A1 and that A549 does not express detectable ALDH1A1.

ALDH is well known as the enzyme downstream from alcohol dehydrogenase in the major pathways of alcohol metabolism, and it comprises ALDH1A1, ALDH2, and ALDH1B1. Of these, ALDH1A1 is known to catalyze the oxidation of retinaldehyde to retinoic acid. Recent studies have proven that ALDH1A1 plays a key role in various biological behaviors in malignant tumors, such as cell proliferation, invasion, and chemoresistance, and it is considered as one of the progenitor markers for stem cells in many different types of tumors [16], [17]. Recently, ALDH1A1 was confirmed to be involved in many pathways regulating biological processes associated with tumor cells [18]–[20]. ALDH1A1 was also found to be important in lung cancers, suggesting an important role in lung tumors [8], [21].In this study, we verified the expression of ALDH1A1 in A549s cells and showed its markedly lower relative expression in A549 cells, where its expression is negligible. Moreover, when A549s cells were treated with ALDH1A1siRNA, their clonogenicity was significantly suppressed, and the proportion of cells in G1 significantly decreased. These results suggested that ALDH1A1 inhibits cell proliferation and improves the clonogenicity of A549s cells.

The Notch pathway is widely used in vertebrate and invertebrate animals. The four subtypes of Notch genes encode four subtypes, comprising a family of transmembrane proteins involved in many cellular processes, including differentiation, proliferation, and apoptosis. Notch can regulate the differentiation and development of cells, tissues, and organs via cell-cell communication. Notch signaling plays a critical role in maintaining the balance between differentiation and proliferation. Notch genes have been shown to directly activate cyclin and CDK2, which can regulate the cell cycle control and affect cellular proliferation [22]. In T-cell lymphoblastic leukemia, the anti-proliferation and apoptotic effects of Notch-1 downregulation may be mediated through regulation of Akt activity and of the expression of the cell cycle regulatory proteins cyclin D1, CDK2, and p21 [23]. The G1–S phase transition is a major checkpoint for cell cycle progression, and CDK2 serves as a critical mediator during this transition [24]. Many drugs can induce apoptosis of cancer cells, but G1 arrest in cancer cells results in their chemoresistance. Li et al. reported that CMTM7, a tumor-suppressor gene, encodes a protein that suppresses tumor growth and which is associated with G1/S cell-cycle arrest through upregulation of p27 and downregulation of CDK2 and CDK6 [25]. Similar results were found for miRNA-342, which inhibits colorectal cancer cell proliferation in vitro [26].

Conversely, Notch inhibits cell development by the induction of cell cycle arrest and apoptosis [27]. To date, Notch activity and its associated downstream signaling are only partially understood. In this study, when we removed Notch expression using siRNA, the expression levels of ALDH1A1, CDK2, and CCNE1 were significantly suppressed, suggesting that the Notch pathway may upregulate the CDK2/cyclin pathway and may potentially promote cell cycle progression in LASCs. In the study, we also found that the expression level of CRIF1 increased when Notch3 was downregulated, and previous study in our lab found that CRIF1 might be associated with the cell cycle arrest of leukemia cells [28], and it might interact with CDK2 to regulate bone marrow microenvironment-induced G0/G1 arrest of leukemia cells(data not shown). Moreover, the results from administration of CDK2 confirmed our hypothesis that the Notch/CDK2/CCNE1 pathway regulates the cell cycle and cell proliferation in LASCs.

In addition to being considered a stem-cell marker, ALDH1A1 has been closely linked with Notch3 [7]. The property may be associated with the cell cycle or with chemoresistance of stem cells [29]. In this study, we chose to evaluate the Notch3 pathway, and we assessed the effects of Notch3 suppression on ALDH1A1expression, clonogenicity, and the cell cycle in A549s cells. Expression of ALDH1A1 and the *Notch* genes were confirmed to be higher in A549s than in A549 cells, as reported previously [30]. When ALDH1A1 was downregulated in A549s cells, the expression levels of all *Notch* genes increased significantly, especially those of Notch1 (by over 15-fold). When ALDH1A1 was overexpressed in A549 cells, the expression levels of all *Notch* genes decreased significantly. Similarly, the expression levels of CDK2 and CCNE1 were changed in parallel with those of *Notch* genes upon modulation of ALDH1A1 expression. This may suggest that ALDH1A1 can inhibit the cell cycle by suppressing the Notch/CDK2/cyclin pathway. Because ALDH1A1 was expressed at high levels in A549s cells, but at low levels in A549 cells, and because ALDH1A1 was able to suppress the Notch/CDK2/CCNE1 pathway, we concluded that ALDH1A1 can inhibit the proliferation of stem cells and maintain their immature state.

Self-renewal, low differentiation, G1 arrest, high chemoresistance, and the ability to differentiate into cancer cells are the basic characteristics of CSCs, which make these cancer cells quite difficult to destroy. ALDH1A1 appears to be one of the main mechanisms for maintaining these characteristics in CSCs. In this study, we isolated A549s cells from A549 cells and confirmed their stemness by multi-directional differentiation. Next, we found that ALDH1A1siRNA inhibited clonogenicity and cell-cycle progression in A549s cells. We further verified that ALDH1A1 can suppress the Notch/CDK2/Cyclin pathway and eventually inhibit the proliferation of these cells.

## Conclusions

ALDH1A1 overexpression improved the clonogenicity of A549s and inhibited the cell cycle, thereby maintaining the stemness of these cells. Furthermore, we showed that this may be mediated via suppression of the Notch/CDK2/Cyclin pathway.
